# N-Doped Graphene Quantum Dots/Titanium Dioxide Nanocomposites: A Study of ROS-Forming Mechanisms, Cytotoxicity and Photodynamic Therapy

**DOI:** 10.3390/biomedicines10020421

**Published:** 2022-02-10

**Authors:** Pravena Ramachandran, Boon-Keat Khor, Chong Yew Lee, Ruey-An Doong, Chern Ein Oon, Nguyen Thi Kim Thanh, Hooi Ling Lee

**Affiliations:** 1 Nanomaterials Research Group, School of Chemical Sciences, Universiti Sains Malaysia (USM), Gelugor 11800, Penang, Malaysia; pravena@ioioleo.com; 2 School of Pharmaceutical Sciences, Universiti Sains Malaysia (USM), Gelugor 11800, Penang, Malaysia; khorboonkeat@student.usm.my (B.-K.K.); chongyew@usm.my (C.Y.L.); 3 Institute of Analytical and Environmental Sciences, National Tsing Hua University, Hsinchu 30013, Taiwan; radoong@mx.nthu.edu.tw; 4 Institute for Research in Molecular Medicine (INFORMM), Universiti Sains Malaysia (USM), Gelugor 11800, Penang, Malaysia; chern.oon@usm.my; 5 Biophysics Group, Department of Physics and Astronomy, University College London, Gower Street, London WC1E 6BT, UK; 6 UCL Healthcare Biomagnetics and Nanomaterials Laboratories, 21 Albemarle Street, London W1S 4BS, UK; 7 School of Chemistry, The University of Sydney, Sydney, NSW 2006, Australia

**Keywords:** titanium dioxide, N-doped graphene quantum dots, photodynamic therapy, near-infrared light, reactive oxygen species, apoptosis

## Abstract

Titanium dioxide nanoparticles (TiO_2_ NPs) have been proven to be potential candidates in cancer therapy, particularly photodynamic therapy (PDT). However, the application of TiO_2_ NPs is limited due to the fast recombination rate of the electron (e^−^)/hole (h^+^) pairs attributed to their broader bandgap energy. Thus, surface modification has been explored to shift the absorption edge to a longer wavelength with lower e^−^/h^+^ recombination rates, thereby allowing penetration into deep-seated tumors. In this study, TiO_2_ NPs and N-doped graphene quantum dots (QDs)/titanium dioxide nanocomposites (N-GQDs/TiO_2_ NCs) were synthesized via microwave-assisted synthesis and the two-pot hydrothermal method, respectively. The synthesized anatase TiO_2_ NPs were self-doped TiO_2_ (Ti^3+^ ions), have a small crystallite size (12.2 nm) and low bandgap energy (2.93 eV). As for the N-GQDs/TiO_2_ NCs, the shift to a bandgap energy of 1.53 eV was prominent as the titanium (IV) tetraisopropoxide (TTIP) loading increased, while maintaining the anatase tetragonal crystal structure with a crystallite size of 11.2 nm. Besides, the cytotoxicity assay showed that the safe concentrations of the nanomaterials were from 0.01 to 0.5 mg mL^−1^. Upon the photo-activation of N-GQDs/TiO_2_ NCs with near-infrared (NIR) light, the nanocomposites generated reactive oxygen species (ROS), mainly singlet oxygen (^1^O_2_), which caused more significant cell death in MDA-MB-231 (an epithelial, human breast cancer cells) than in HS27 (human foreskin fibroblast). An increase in the N-GQDs/TiO_2_ NCs concentrations elevates ROS levels, which triggered mitochondria-associated apoptotic cell death in MDA-MB-231 cells. As such, titanium dioxide-based nanocomposite upon photoactivation has a good potential as a photosensitizer in PDT for breast cancer treatment.

## 1. Introduction

In recent years, scientists have focused on nanoparticle research in the biomedical field, particularly in cancer therapies. Nanoparticles offer numerous advantages as they can easily penetrate tissues, cross cellular barriers, preferentially localize and accumulate at tumor sites and are able to overcome instant clearance by the lymphatic system [1]. Nanoparticles can play multiple roles, as they can be used for diagnosis and therapy simultaneously. Metal or metal oxide nanoparticles can generate ROS in the presence of light illumination to induce cell death [2,3]. Acting as a photosensitizer or nanocarrier, metal oxides exhibit relatively good stability when compared to existing organic nanoparticles (liposomes, dendrimers, polymer-based NPs), with regard to temperature and pH change [4,5].

An optical irradiation-induced generation of ROS by a photosensitizer that promotes cell killing is known as photodynamic therapy (PDT). PDT is an emerging non-invasive, clinically approved and localized therapy for several diseases, including cancers. PDT surpasses existing traditional cancer treatments as it can be specifically targeted, is non-invasive, causes negligible drug resistance and is highly effective with fewer adverse side effects [6].

The efficacy of PDT depends on the type of photosensitizing agents employed. Numerous inorganic and organic materials such as cadmium selenide (CdSe), chlorin e6 (Ce6), hypocrellin A (HA), inorganic [7] and porphyrin-based materials and organic materials [8], were explored as photosensitizing agents in PDT for cancer treatments. However, most of these materials have drawbacks including poor water dispersibility and photostability, as well as the inability to absorb at longer wavelengths (>700 nm), which restricts light penetration leading to the imprecision of their cell-killing potential. This leads to undesirable toxicity, potentially causing damage to both cancer and non-cancerous cells/tissues.

The application of metal oxide nanoparticles as a photosensitizer has been extensively studied due to the limitations of existing porphyrin-based photosensitizers. Upon administration via tumoral injection, nanoparticle-based drugs are preferentially taken up and accumulate at the tumor sites due to the enhanced permeability and retention (EPR) effect, a condition where tumors possess unusually leaky blood vessels and an impaired lymphatic system [9]. Thus, it offers an effective method to precisely locate and cause tumor cell destruction simultaneously, preventing overdose of photosensitizers with controllable treatment duration.

Among the existing metal oxide nanoparticles, TiO_2_ NPs have attracted considerable research interest due to their unique photocatalytic properties that can be utilized to kill cancer cells upon illumination. When TiO_2_ NPs are irradiated with an energy equal to or greater than the bandgap of TiO_2_ (3.2 eV), the electrons (e^−^) in the valence band (VB) of TiO_2_ are excited to the conduction band (CB), creating positive holes (h^+^) in the VB. This occurrence leads to a redox reaction on the surface of these semiconductor nanoparticles resulting in the generation of ROS, comprising of superoxide anions (O_2_^−•^), hydroxyl radicals (^•^OH) and hydrogen peroxide (H_2_O_2_) [10,11].

Meanwhile, as an inorganic photosensitizer, TiO_2_ is more stable than classic photosensitizers in performing PDT. This trait is attributed to the nanoscale size and anti-photodegradable stability of TiO_2_. It has been reported that TiO_2_ NPs were used as a PDT agent in various types of cancer cells, such as human hepatocellular carcinoma cell line (HepG2) [12], leukemia cells (K592) [13], cervical cancer cells (HeLa) [14], breast epithelial cells (MCF7 and MDA-MB-468) [15], as well as non-small cell lung cancer (NSCLC) [16]. Despite their excellent performance as photosensitizers, their potential toxicity is still an obstacle to their application in PDT [17,18]. Furthermore, the activation of pristine TiO_2_ is triggered upon shorter wavelength UV light irradiation to generate ROS.

To overcome the shortcomings of TiO_2_, depositing quantum dots onto TiO_2_ has received the utmost attention due to their distinctive properties. Modifying the surface properties of TiO_2_ with QDs will assist in extending the light absorption properties of TiO_2_ to longer wavelengths. Therefore, this will allow deeper penetration into tissues. TiO_2_ modified with transition metals (Cu, Zn) and metal oxides (ZnO, NiO) often induce additional ROS generation in the absence of light irradiation. Moreover, incorporating noble metals such as Ag and Pt is considered to be more effective due to their high stability and anti-cancer activity, but their high cost restricts the application [19,20,21,22,23,24]. Generally, in PDT, QDs possess a dual-function nature as an energy transducer and as carriers of photosensitizers [25]. Sensitizing TiO_2_ with metal chalcogenides quantum dots such as CdSe, cadmium sulfide (CdS), lead sulfide (PbS), cadmium telluride (CdTe) and copper oxide (CuO) inevitably impede their benefits [26,27,28,29,30]. Most of these QDs of group II–VI contain highly toxic heavy metals that are unstable and induce toxicity, which causes environmental hazards. Thus, incorporating TiO_2_ with carbonaceous-material-based QDs has been identified as a potential approach. Therefore, the utilization of N-GQDs to modify TiO_2_ surfaces has great research interest. Besides, heteroatom doping (N atom) of GQDs results in high quantum yield, good stability, and higher catalytic activity, by tuning their electrochemical properties [31]. Good biocompatibility properties of N-GQDs will help to reduce the toxicity of TiO_2_ in the absence of light illumination, which warrants further exploration in PDT as a photosensitizer [32]. Furthermore, as a carrier, N-GQDs ensure precise localization of the potential N-GQDs/TiO_2_ NCs at the site of the tumor and, therefore, avoid harm to non-cancerous cells. Thus, the incorporation of TiO_2_ with N-GQDs holds great promise in PDT.

Herein, in this study, TiO_2_ NPs and TiO_2_ conjugated with N-GQDs (N-GQDs/TiO_2_ NCs) were synthesized via microwave-assisted synthesis and two-pot hydrothermal method, respectively. Their corresponding in vitro cytotoxicity was studied using the MDA-MB-231 Triple-negative breast cancer (TNBC) breast cancer cell line and HS27 human fibroblast cell line to evaluate the cellular response towards the nanostructures. TNBC is an aggressive subtype with a poor prognosis. In order to assess the impact of the nanocomposite, we have chosen a more aggressive type than the conventional MCF-7 cells. Although chemotherapy remains the mainstay for the treatment of TNBC, systemic toxicity and adverse effects associated with chemotherapy highlight the need for an alternative theory. As such, MDA-MB-231 TNBC (mentioned as MDA-MB-231 from now onward) was employed as the cell model to be evaluated in the current proposed photodynamic therapy. The photodynamic activity of the near-infrared light active-N-GQDs/TiO_2_ NCs was evaluated by monitoring the in vitro photokilling effects on the cells under light irradiation and the mechanisms where the nanocomposites possessed photokilling properties were investigated.

## 2. Materials and Methods

### 2.1. Materials

Citric acid-1-hydrate (Bendosen, Laboratory Chemicals, Johor Bahru, Malaysia); ethylenediamine; glycerol; ethanol, 98%; hydrochloric acid, HCl 37%; phosphate-buffered saline (PBS) (QREC, Grade AR, (Asia) Sdn. Bhd, Rawang, Selangor, Malaysia); titanium(IV) tetraisopropoxide, TTIP ≥ 97% purity; commercial pure anatase, ≥99%; sodium pyruvate; dimethyl sulfoxide, DMSO; 1,4-benzoquinone (Sigma-Aldrich, Co., St. Louis, MO, USA); phosphate buffered saline (PBS) (Sigma-Aldrich, Co., Taufkirchen, Germany); Dulbecco’s modified Eagle’s medium, DMEM; penicillin-streptomycin solution, 10,000 units/mL (Nacalai Tesque, Nakagyo-ku, Kyoto, Japan); fetal bovine serum (FBS) (TICO Europe, DJ, Amstelveen, The Netherlands); CellTiter 96^®^ AQueous One Solution Cell Proliferation Assay (MTS); Caspase-Glo^®^ 3/7 Assay (Promega, Madison, WI, USA); 2′,7′-dichlorofluorescein diacetate (DCFDA, Merck-Millipore, Burlington, MA, USA); tetramethylrhodamine ethyl ester; TMRE-Mitochondrial Membrane Potential Assay Kit (Abcam, Trumpington, Cambridge CB2 0AX, UK) were used as purchased without any further purification.

### 2.2. Synthesis of TiO_2_ NPs

The TiO_2_ NPs were prepared according to the methodology developed by our laboratory [32]. Briefly, 2 mL of TTIP was hydrolyzed and stirred vigorously at room temperature. The pH of the homogenous mixture was adjusted to 1.3 by adding 37% hydrochloric acid with constant stirring for 30 min. The entire mixture was then transferred to a 100 mL sealed vessel made of high-purity TFM (modified Teflon), which was heated in the commercial microwave digestion system (Multiwave 3000 Anton Paar, Graz, Austria) at 600 W for 20 min. The resulting precipitate was washed with distilled water for several cycles and isolated through centrifugation (8500 rpm, 10 min), followed by drying in the oven at 50 °C for 24 h. The dried TiO_2_ NPs were annealed at 500 °C for 2 h.

### 2.3. Synthesis of N-GQDs/TiO_2_ NCs

The N-GQDs/TiO_2_ NCs were synthesized using the methodology developed previously [32]. Briefly, 3 mL of TTIP in 50 mL distilled water was continuously stirred for 30 min before transferring into a Teflon-lined stainless-steel autoclave (capacity = 100 mL). The mixture in the stainless hydrothermal reactor was heated in the oven at 160 °C for 24 h. The obtained sol was washed in distilled water and placed in a beaker containing 50 mL distilled water. Then, citric acid and ethylenediamine were added simultaneously into the magnetically stirred sol for 30 min, followed by hydrothermal treatment at 180 °C for 4 h. The obtained precipitate was washed, centrifuged and dried in a vacuum oven at 60 °C overnight.

### 2.4. Characterizations

The powder X-ray diffraction (XRD) technique was used to analyze the crystallographic structure, crystallinity and phase purity of the synthesized samples. The XRD patterns were recorded on a PW 3040/60 X’PERT PRO, PANalytical using CuKα (1.5406 Å) radiation in the range 2θ = 10–90°. Besides, X-ray photoelectron spectroscopy (XPS) was used to identify the surface composition of the synthesized materials. A High-Resolution Multi Technique X-Ray Spectrometer (Axis Ultra DLD XPS, Kratos) with monochromatic Al Kα (1486.6 eV), X-ray radiation (15 kV and 10 mA) and equipped with a hemispherical analyzer which operated at 150 W was used to analyses the materials. Curve fitting was accomplished using OriginPro (version 8.5), whereby all the obtained binding energy (BE) was calibrated using the C 1s line at 284.6 eV. Meanwhile, a Perkin Elmer Lamda 35 was used to record ultraviolet-visible diffuse reflectance (UV-Vis DRS) spectra of the samples.

### 2.5. Cell Culture and the Conditions

MDA-MB-231 was obtained from Dr. Chern Ein Oon (INFORMM, USM); meanwhile, HS27 was obtained from the Centre for Drug Research, USM. The MDA-MB-231 and HS27 cells were cultured in DMEM medium and DMEM high glucose medium, respectively, were then supplemented with 10% FBS and 1% penicillin–streptomycin, and incubated at 37 °C with 5% CO_2_ and 90% humidity.

### 2.6. Cytotoxicity Assay

The in vitro cytotoxicity assay of nanomaterials was performed using an MTS assay following the manufacturer’s protocol. The cells were seeded in 96-well plate (10,000 cells/well) and treated with cell culture medium containing different concentrations of nanomaterials (0.01, 0.05, 0.1, 0.5 and 1.0 mg mL^−1^) for 24 h. Then, the MTS solution was added to each well, followed by incubation for 4 h. The absorbance of each well at 490 nm was recorded using a Multiskan Go UV microplate reader (ThermoFisher Scientific, Waltham, MA, USA). The cells that were incubated with cell culture medium without any treatment were referred to as the control group. The results are expressed in mean ± standard deviation (SD) as a percentage compared to control. The cell viability was calculated according to the following Equation (1).

(1) $$\mathrm{Percentage}\;\mathrm{of}\;\mathrm{viable}\;\mathrm{cells}\;\left(\%\right)=\frac{\mathrm{Absorbance}\;\left(\mathrm{Treated}\right)-\mathrm{Absorbance}\;\left(\mathrm{Blank}\right)}{\mathrm{Absorbance}\;\left(\mathrm{Untreated}\right)-\mathrm{Absorbance}\;\left(\mathrm{Blank}\right)}\times 100\%\;$$

### 2.7. Photokilling Effects of N-GQDs/TiO_2_ NCs on MDA-MB-231 and HS27 Cells

To study the photokilling effects, cells seeded in 96-well plates were incubated with a medium containing 0.05–0.5 mg mL^−1^ N-GQDs/TiO_2_ NCs for 3 h in the dark. Then, the cells were washed with PBS and re-suspended in a fresh medium. The cells were irradiated with 20 W tungsten halogen lamp with an ultraviolet and visible cut-off filter eliminating UV-Vis light λ < 700 nm, resulting in NIR (700–900 nm) at 55 mW cm^−2^, which then resulted in light energies of 16.5, 33 and 66 J cm^−2^ for 5, 10 and 20 min, respectively. The light intensity was measured using a Solar Light PMA2100 Dual-Input Data Logging Radiometer. The irradiated cells were then incubated in the dark for 24 h. After the incubation period, the cell viability was determined using the MTS assay. The results are expressed in mean ± SD as a percentage compared to control.

### 2.8. Measurement of Reactive Oxygen Species (ROS) Level

#### 2.8.1. Measurement of Intracellular ROS Levels

The intracellular levels of ROS were measured by exposing the cells to different concentrations of N-GQDs/TiO_2_ NCs for 3 h, were washed with PBS and then re-suspended in a fresh DMEM medium. The cells were then irradiated for 20 min and incubated at 37 °C for 4 h. Five mM of DCFDA was used as a stock, then 5 µL of 5 mM DCFDA was further diluted in 5 mL medium to make up the final concentration of 5 µM. After the incubation, the medium was replaced with 100 µL of 5 µM DCFDA solution and incubated at 37 °C for 1 h. Then, the DCFDA solution was removed, washed with PBS before incubating with 1% Triton-X lysis buffer (100 µL) for 30 min. Thereafter, lysed cells were centrifuged at 10,000 rpm for 15 min. The suspension was harvested and placed into 96-well plates. Clean lysis buffer acted as a blank control and was prepared in parallel with samples. The fluorescent intensity was measured using a Plate Chameleon™ Multitechnology plate reader at excitation and emission wavelengths of 485 nm and 535 nm, respectively. The data are expressed in mean ± SD as a percentage compared to control.

#### 2.8.2. Measurement of Specific Types of ROS

To evaluate the specific types of ROS generated by N-GQDs/TiO_2_ NCs with light irradiation, several ROS quenchers for specific ROS were used, including sodium pyruvate, DMSO, 1,4-benzoquinone and glycerol for the detection of H_2_O_2_, ^•^OH, O_2_^−•^ and ^1^O_2_/O_2_^−•^, respectively. First, 100 µL of 0.5 mg mL^−1^ N-GQDs/TiO_2_ NCs in PBS were mixed with 50 µL DCFDA (without quenchers) and irradiated with light. The fluorescent intensities of DCFDA were recorded, and a linear plot of intensity versus time was plotted with a slope noted as S_ref_. Then, several specific ROS quenchers (10 mM sodium pyruvate, 0.28 M DMSO, 10 mM 1,4-benzoquinone and 5 vol % glycerol) were respectively added into the sample solution containing DCFDA before irradiation [33,34,35]. For the light-irradiated sample solutions, the level of ROS generated in the presence of quenchers was measured with an interval of 5 min for 20 min using a Plate Chameleon TM Multitechnology plate reader at excitation and an emission wavelength of 485 nm and 535 nm, respectively. The slope of the linear line plotted based on the results obtained is expressed as S_q_. The results are expressed in mean ± SD as a percentage compared to control. Thus, the specific ROS percentage was calculated according to the following Equation (2).

(2) $$\mathrm{ROS}\;\mathrm{generated}\;\left(\%\right)=\left(1-\frac{S_{q}}{S_{\mathrm{ref}}}\right)\times 100\%$$

### 2.9. Measurement of Caspase-3/7 Activity

Caspase-3/7 activity in the cells was assessed using Caspase-Glo^®^ 3/7 reagent following the manufacturer’s protocol. The treated and non-treated MDA-MB-231 cells were washed with PBS and re-suspended in a fresh DMEM medium. Thereafter the light irradiation, the cells were incubated for 24 h. A 100 µL of Caspase-Glo^®^ 3/7 reagent was added to each well, followed by 3 h incubation at room temperature in the dark. Non-treatment cells were used as a control in this assay. The luminescence readings were then measured using the M5 multi-detection microplate reader. The data were expressed in mean ± SD as percentage compared to control.

### 2.10. Measurement of Mitochondrial Activity

To further study the mitochondrial activity of the cells after the PDT treatment, a tetramethylrhodamine ethyl ester (TMRE) assay was used following the manufacturer’s protocol. Briefly, 1 µM TMRE (100 µL) was added to each well of the control and PDT-treated MDA-MB-231 cells. Then, the fluorescent readings were then measured using the M5 multi-detection microplate reader at excitation and an emission wavelength of 544 nm and 590 nm, respectively. The data are expressed in mean ± SD as percentage compared to control.

### 2.11. Statistical Analysis

The data are presented as the mean ± SD of two independent experiments, each performed in triplicate. The statistically significant differences in cell viability (*p* < 0.05) were analyzed using an ANOVA by GraphPad Prism 5.0 software, California. The ANOVA test is carried out to compare the statistical differences among different cell groups (control cells, HS27 and MDA-MB-231 cells).

## 3. Results and Discussion

### 3.1. X-ray Powder Diffraction

**Figure 1:**
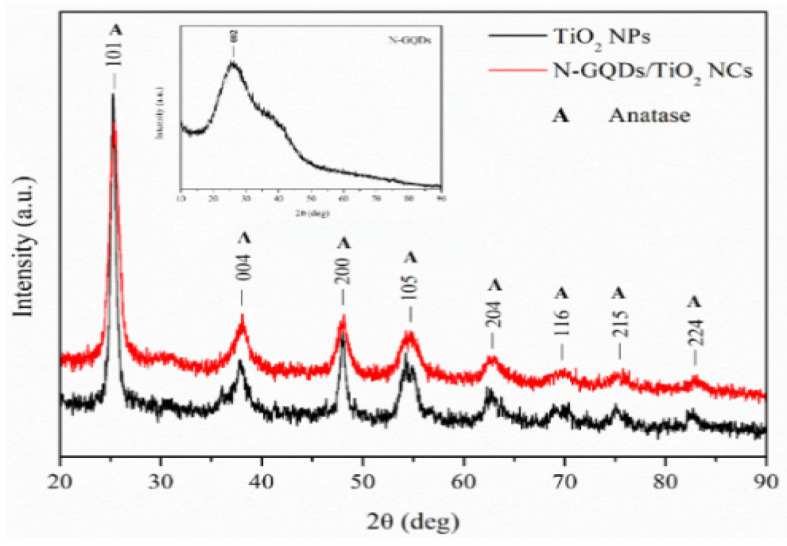
X-ray diffractograms synthesized TiO_2_ NPs and N-GQDs/TiO_2_ NCs. The inset shows the X-ray diffractograms of N-GQDs.

The XRD analysis was executed to study the crystallographic properties of pure TiO_2_ NPs and N-GQDs/TiO_2_ NCs. The XRD patterns of TiO_2_ NPs and N-GQDs/TiO_2_ NCs are depicted in Figure 1. The Figure 1 shows the reflection of (101), (004), (200), (105), (211), (204), (116), (220), (215) and (224) peaks at 2θ values of 25.27°, 37.79°, 48.21°, 54.40°, 55.06°, 62.56°, 68.85°, 70.25°, 75.16° and 82.88°, respectively, which correspond to the formation of the pure single-phase of anatase TiO_2_ NPs with tetragonal structure (space group, I41/amd, lattice parameter, a = b = 0.378 nm and c = 0.951 nm), which are indexed based on ICSD 01-071-1166. Similarly, the diffraction peaks of TiO_2_ incorporated with N-GQDs were in good agreement with the typical diffraction pattern of anatase phase TiO_2_ (ICSD 01-071-1166) and the obtained XRD pattern matched well with as-synthesized TiO_2_ NPs. The (101) planes, prominently reflected in the XRD pattern of the nanomaterials, were in good agreement with the measured lattice spacing of 0.351 nm, as reported in our previous work [32]. The obtained findings suggest that the structure and phase purity of TiO_2_ remained intact, as N-GQDs were incorporated on the surface of the TiO_2_, and not into the TiO_2_ lattice. Moreover, no additional peaks attributed to the N-GQDs were observed in all the nanocomposite samples. This observation could be due to the lower concentration of N-GQDs and relatively low diffraction intensity of graphene (2θ ~ 25.60°) in the nanocomposites, thus, these might be shielded by the major peak in the anatase phase TiO_2_ (2θ = 25.26°) [36]. Besides, it can be observed that the intensity of the diffraction peaks’ height declined with the incorporation of N-GQDs, due to the low crystalline nature of the N-GQDs [37].

Table S1 presents the crystallite size, lattice parameters and lattice strain calculated from XRD data obtained for both samples. The obtained results depict that the average crystallite size (Scherrer equation) of N-GQDs/TiO_2_ NCs decreased when compared to the pure TiO_2_ NPs, which can be attributed to the confinement effect of graphene sheets that were ascribed to the size change in the sp^2^ domains, similar to the reported studies [38,39]. The obtained results are further supported by the average particle size obtained based on the high resolution transmission electron microscopic (HRTEM) analysis (TiO_2_ NPs = 11.46 ± 2.8 nm, N-GQDs/TiO_2_ NCs = 9.16 ± 2.4 nm) [32]. Moreover, the lattice parameters a and c correspond to the respective XRD patterns of the TiO_2_ NPs, and N-GQDs/TiO_2_ NCs were in accordance with the reference data of the anatase tetragonal structure (ICSD 01-071-1166). The incorporation of N-GQDs onto the surface of TiO_2_ poses no influence on the lattice parameters. Meanwhile, the calculated lattice strain of N-GQDs/TiO_2_ NCs was higher than that of TiO_2_ NPs, due to the smaller crystallite size and a lower degree of crystallinity, ascribed to the lower crystalline nature of N-GQDs in the nanocomposite [40]. Thus, conjugation of N-GQDs into TiO_2_ lowers the crystallinity and particle size, which induces additional strain without altering the lattice parameter values.

### 3.2. UV-Visible Diffuse Reflectance Spectroscopy (UV-Vis DRS)

**Figure 2:**
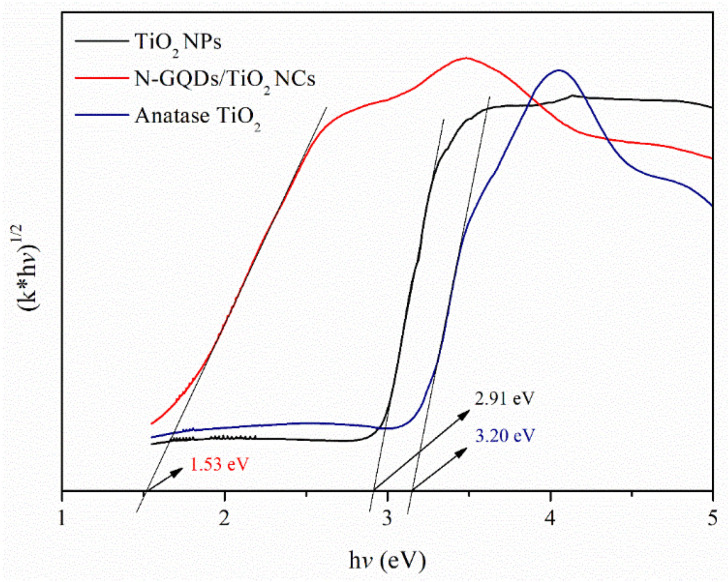
The bandgap of synthesized TiO_2_ NPs, N-GQDs/TiO_2_ NCs and anatase TiO_2_.

The calculated bandgap curves of TiO_2_ NPs and N-GQDs/TiO_2_ NCs are shown in Figure 2. The spectrum of anatase TiO_2_ is also shown for better comparison. The bandgaps were obtained based on the Kubelka-Munk rule by plotting (khv)^1/2^ versus photon energy (hv). Based on the results obtained, it was found that all N-GQDs incorporated into TiO_2_ recorded lower bandgap energies than that of synthesized TiO_2_ (2.91 eV) and commercial pure anatase TiO_2_ (3.20 eV). The reduction in the bandgap of the as-synthesized TiO_2_ NPs is attributed to surface defects due to the presence of surface oxygen vacancies and Ti^3+^ self-doping, which was further confirmed by XPS analysis in Section 3.3. Moreover, the bandgap value of TiO_2_ NPs reflects that it can be activated upon the visible light source. Besides, as no Ti^3+^ environment was found in the Ti 2p spectra of the nanocomposite (Section 3.3), therefore the significant blue-shift in the bandgap energy of the N-GQDs/TiO_2_ NCs is attributed to the existence of Ti-O-C bonding between N-GQDs and TiO_2_ [41,42]. This interaction facilitates an efficient interfacial charge transfer process between TiO_2_ and N-GQDs. This phenomenon further ensures the prolonged lifetime of the excited states due to the improved charge separation in the nanocomposites. This observation implies that the introduction of N-GQDs onto the surface of TiO_2_ has shifted the optical bandgap, which enables the nanocomposite to generate e^−^/h^+^ pairs even though they have been irradiated with longer, non-toxic NIR light.

### 3.3. X-ray Photoelectron Spectroscopy (XPS)

**Figure 3:**
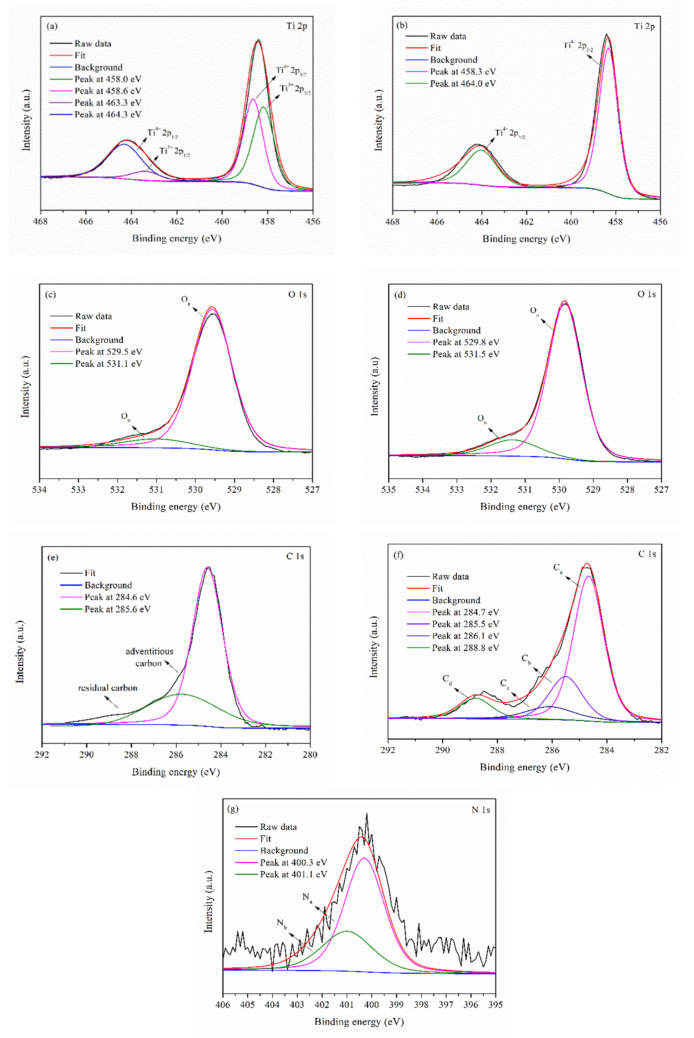
High-resolution XPS spectra of Ti 2p of synthesised (**a**) TiO_2_ NPs and (**b**) N-GQDs/TiO_2_ NCs, O 1s of synthesized (**c**) TiO_2_ NPs and (**d**) N-GQDs/TiO_2_ NCs, C 1s of synthesised (**e**) TiO_2_ NPs and (**f**) N-GQDs/TiO_2_ NCs, (**g**) N 1s of synthesized N-GQDs/TiO_2_ NCs.

XPS was employed to study the surface composition and chemical states of TiO_2_ NPs and N-GQDs/TiO_2_ NCs. The resulting high-resolution XPS spectra depict Ti 2p, O 1s and C 1s states for both samples. Meanwhile, an additional XPS spectrum of N 1s was recorded for N-GQDs/TiO_2_ NCs. The Ti 2p spectrum of both samples show two prominent peaks at ~458.0 eV and ~464.0 eV, assigned to Ti 2p_3/2_ and Ti 2p_1/2_ spin-orbital splitting photoelectrons, respectively (Figure 3a,b). It was found that there was a blue-shift of 0.3 eV in both Ti^4+^ 2p_3/2_ (458.3 eV) and Ti^4+^ 2p_1/2_ (464.0 eV) peaks in the N-GQDs/TiO_2_ NCs when compared with those in the synthesized TiO_2_ spectrum (Ti^4+^ 2p_3/2_ = 458.6 eV and Ti^4+^ 2p_1/2_ = 464.3 eV). This shifting might be due to the formation of Ti-O-C bonds [43,44]. Besides, further deconvolution of Ti 2p of TiO_2_ NPs resulted in another two peaks which are Ti^3+^ 2p_3/2_ (458.0 eV) and Ti^3+^ 2p^1/2^ (463.3 eV), while no Ti^3+^ state was observed in N-GQDs/TiO_2_ NCs. With the appearance of the Ti^3+^ state, it suggests the as-synthesized TiO_2_ NPs are self-doped TiO_2_. Enhanced microwave power irradiation could lead to the conversion of Ti^4+^ to Ti^3+^ by forming oxygen vacancies. Generally, microwave energy can increase the heating rate. Thus, conducting the reaction at higher microwave power will result in a higher rate of hydrolysis and condensation of the TTIP precursor [45]. Besides, as the condensation rate increased, it increases the formation of oxygen vacancy and Ti^3+^ ions by removing more surface oxygen. Meanwhile, the excess electrons from oxygen vacancies are trapped on Ti^4+^ ions to form Ti^3+^ species. The proposed formation mechanism of oxygen vacancies and Ti^3+^ ions is shown in Figure S1.

Whereas, for the O 1s spectra (Figure 3c,d), it was found that several chemical states of oxygen were present in the samples. Both samples exhibited the main peak centered at 529.0 eV, assigned to the lattice oxygen (Ti-O-Ti). Another peak, observed at 531.1 eV for TiO_2_ NPs, corresponds to the hydroxyl group that adsorbed on the surface of the TiO_2_ [45,46]. Additionally, a peak at 531.5 eV is assigned to the Ti-O-C bonds in N-GQDs/TiO_2_ NCs, suggesting that the N-GQDs and TiO_2_ were probably coupled via Ti-O-C bonds, which could promote interfacial electron transfer [47,48].

Meanwhile, the C 1s spectra of TiO_2_ NPs and N-GQDs/TiO_2_ NCs (Figure 3e,f) were deconvoluted and fitted with two and four peaks, respectively. A strong peak at 284.6 eV and a shoulder peak at 285.6 eV in TiO_2_ NPs are attributed to the adventitious carbon of the carbon tape attached to the sample holder and residual carbon that was associated with the carbon residues from the TTIP precursor, respectively [43,49]. Furthermore, the peak at 284.7 eV in N-GQDs/TiO_2_ NCs was assigned to sp^2^ hybridized carbon atoms (C=C) in the honeycomb lattice structure of N-GQDs.

Moreover, three peaks, centered at 285.5 eV, 286.1 eV and 288.8 eV, were ascribed to a C-N bond with sp^2^ orbital, C-OH (hydroxyl carbon) and O-C=O (carboxylate carbon), respectively. There was no Ti-C carbide bond-related peak (~282 eV) observed in the C 1s spectrum of N-GQDs/TiO_2_ NCs [50]. This finding further implies the anchoring of N-GQDs on the surface of TiO_2_ via Ti-O-C bond formation.

Furthermore, an additional N 1s spectrum was observed for N-GQDs/TiO_2_ NCs and it was fitted into two peaks (Figure 3g). The main peak at 400.3 eV is attributed to the pyrrolic N and the peak at 401.1 eV corresponds to graphitic N within the graphene lattice [37,51]. The absence of a Ti-N bond (~396 eV) in the N 1s spectrum indicates that TiO_2_ was not doped with N atoms, while it reaffirms the presence of N atoms doped into the graphene lattice [52].

### 3.4. Characterisation of the Nanomaterials in Cell Culture Treatment, Their In Vitro Cytotoxicity Assessment and Photodynamic Therapy

The behavior of the nanomaterials in the cell culture environment and their interaction with biological substrates were studied by determining their respective hydrodynamic size and zeta potential in the deionized water (DI water) and cell culture medium (Supplementary Figure S2). Overall, nanomaterials dispersed in DMEM without any dispersing agent resulted in a higher hydrodynamic size than in DI water, due to higher ionic strength content in the cell culture medium. Meanwhile, there was a significant decrease in the hydrodynamic size of the nanomaterials dispersed in complete cell culture medium (DMEM + 1% FBS). This effect is attributed to the formation of protein corona, which provides electrostatic repulsion between particles. These findings are in good agreement with previously reported works [13,20]. Furthermore, hydrodynamic sizes of 0.1 mg mL^−1^ N-GQDs/TiO_2_ NCs (49.2 ± 4.5 nm) dispersed in complete cell culture medium were observed to be smaller than for TiO_2_ NPs (51.1 ± 3.3 nm). Consistent with the obtained hydrodynamic size, the zeta potential values of 0.1 mg mL^−1^ N-GQDs/TiO_2_ NCs (−23.2 ± 2.1 mV) in a medium containing 1% FBS were more negatively charged than for TiO_2_ NPs (−21.5 ± 1.6 mV), suggesting nanocomposite disaggregation. Moreover, N-GQDs (0.1 mg mL^−1^) dispersed in complete cell culture medium have the smallest hydrodynamic size (11.8 ± 5.2 nm) and a large negative value of zeta potential (−30.0 ± 2.7 mV), which leads to good dispersion of the quantum dots. These properties of N-GQDs improve the dispersibility of the TiO_2_ in the nanocomposite [32].

In this study, the in vitro cytotoxicity of TiO_2_ NPs and N-GQDs/TiO_2_ NCs (0.01, 0.05, 0.1, 0.5 and 1.0 mg mL^−1^) were evaluated using MDA-MB-231 and HS27 cells for 24 h.

**Figure 4:**
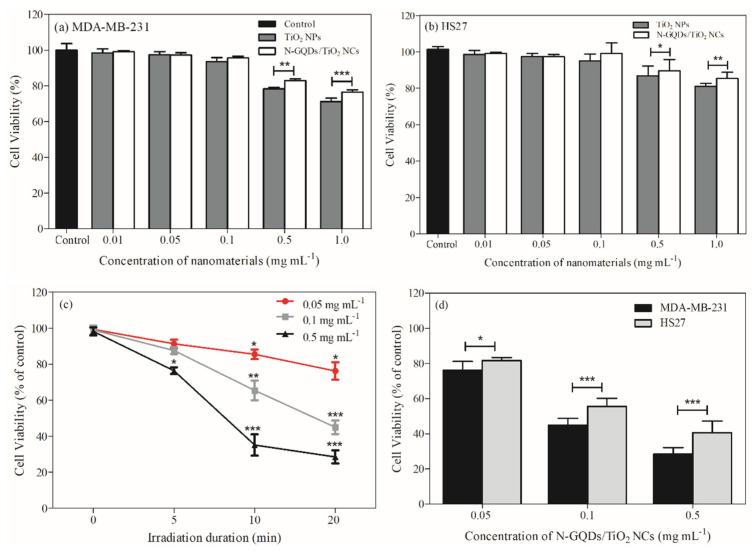
Cell viability of (**a**) MDA-MB-231 and (**b**) HS27 cells, (**c**) MDA-MB-231 cells treated with different concentrations of N-GQDs/TiO_2_ NCs (0.05–0.5 mg mL^−1^), then irradiated with various doses of NIR light (5–20 min) and (**d**) MDA-MB-231 and HS27 cells treated with different concentrations of N-GQDs/TiO_2_ NCs (0.05–0.5 mg mL^−1^), then irradiated with NIR light for 20 min. The cell viability was estimated at 24 h after irradiation. Data are presented as the mean ± SD of two independent experiments made in three replicates (*n* = 6). Significant difference was tested using one and two-way ANOVA followed by the Tukey’s (**a**,**b**,**d**) and Bonferroni post-hoc tests (**c**), respectively * (*p* < 0.05), ** (*p* < 0.01) and *** (*p* < 0.001).

The reason we conducted the PDT reaction for 24 h was due to the significant drop in the cell viability after 48 h treatment when compared to 24 h without light irradiation, based on our previous study [32]. Cytotoxicity of nanocomposites in the absence of light is a critical property before PDT treatment. Similarly, other work also studied the 24 h PDT of TiO_2_ that was conjugated with reduced graphene oxide [12]. The MDA-MB-231 cells were used as a cancer cell model, while HS27 represented a non-cancerous cell, control model to test the cytotoxicity of the nanomaterials. As shown in Figure 4a,b, the viability of the cells after 24 h incubation was not significantly altered as the concentration of TiO_2_ NPs increased from 0.05 to 0.5 mg mL^−1^, then, at 1.0 mg mL^−1^, there was a 29% and 23% decrease in viability of MDA-MB-231 and HS27 cells, respectively. Moreover, when compared to the control group, the lower concentrations of nanoparticles (0.05–0.1 mg mL^−1^) did not exhibit a significant growth inhibitory effect in the cell viability of both cell lines. Moreover, the cell viability trend of the synthesized nanocomposites was similar to that of TiO_2_ NPs at lower concentrations (0.01–0.1 mg mL^−1^). However, it increased significantly at 0.5 and 1.0 mg mL^−1^ when compared to TiO_2_ NPs in both cell lines. Furthermore, this observation indicates that TiO_2_ NPs exhibit a more prominent toxicity level than the nanocomposites (0.5 mg mL^−1^ (*p* < 0.01) & 1.0 mg mL^−1^ (*p* < 0.001)), which is attributed to their distinct characteristics (particle size, crystallinity and composition) in both cell lines [53,54]. Meanwhile, as for the nanocomposite, good biocompatibility characteristics of N-GQDs (0.5 mg mL^−1^ (*p* < 0.05) & 1.0 mg mL^−1^ (*p* < 0.01)) after 24 h post-treatment helps to mitigate the toxicity effects of TiO_2_ (Supplementary Figure S3). Based on the obtained results, it has been proven that incorporating N-GQDs into TiO_2_ did not render any additional toxicity to the nanocomposite when compared to TiO_2_ NPs after 24 h post-treatment [32]. The initial cell viability study presents the safe concentration from 0.01 mg mL^−1^ to 0.5 mg mL^−1^, as viability decreased prominently at 1.0 mg mL^−1^. Overall, the synthesized nanomaterials induced toxicity in a dose- and time-dependent manner.

The ‘safe concentrations’ of the nanocomposite that were identified from the cytotoxicity assay were used to determine their photokilling properties on MDA-MB-231. The cell-killing effects were measured under irradiation of near-infrared (NIR) light for 5, 10 and 20 min resulting in light energies of 16.5, 33 and 66 J cm^−2^, respectively, as shown in Figure 4c. A low-power (20 W) lamp was used in this study to minimize the effect of temperature. The nanocomposite led to a slight decrease in cell viability at lower concentrations (0.05 mg mL^−1^) and short irradiation duration (5 min) when compared to the control cells. However, a substantial decrease in cell viability with the highest photokilling effect was observed at nanocomposite doses of 0.1 and 0.5 mg mL^−1^ under the irradiation of NIR light for 20 min. Irradiation of nanocomposites (0.05–0.5 mg mL^−1^) for 20 min resulted in a 24, 65 and 72% reduction in cell viability, respectively, when compared to control cells as determined after 24 h of incubation. It is noteworthy that, unlike carcinogenic UV light, NIR light is generally considered safe to humans, without inducing adverse side effects such as tissue damage, severe skin aging or oxidative stress [55]. Besides, the application of longer wavelength NIR light is more effective in PDT than UV and visible light as it has a greater ability to penetrate the human skin, and reaches the subcutaneous tissues (e.g., deeper-seated tumor), the capillaries and other major components of living tissues such as water, hemoglobin in blood and proteins [56]. The obtained results indicated that both the applied concentration of N-GQDs/TiO_2_ NCs and light irradiation duration, as well the intensity, regulate the induction of cell death. Moreover, the anti-cancer effects of N-GQDs/TiO_2_ NCs are light exposure-dependent.

The efficacy of the treatment for cancer cells mainly depends on cellular selectivity and inducing photodamage to the targeted cancer cells when compared to normal cells. To test the selectivity of the treatment, a comparative study was carried out on the HS27 cell line, since the human skin will be the first to be exposed to the light-based PDT treatment before deep penetration into the breast cancer cells (Figure 4d). Similar to the results obtained in the cytotoxicity assay, a lower concentration (0.05 mg mL^−1^) of nanocomposites had no significant influence on the photokilling effects on HS27. When nanocomposite concentrations of 0.1 and 0.5 mg mL^−1^ were used, a 45 and 60% reduction in HS27 cells were observed, respectively. Nevertheless, the obtained finding of HS27 cells was less than the 65% and 72% reduction seen in the cell viability of MDA-MB-231 cells at the respective concentrations of the nanocomposite. The phototoxic effect of PDT was observed in both non-cancerous and cancer cells, however, synthesized N-GQDs/TiO_2_ NCs selectively induced more photodamage in the cancerous MDA-MB-231 cells than HS27 cells. The selective toxicity of the nanocomposite might be associated with the difference in the morphology [57], as well as structural and functional differences, of the mitochondria [58,59] between cancer and normal cells.

### 3.5. Production of Reactive Oxygen Species (ROS)

**Figure 5:**
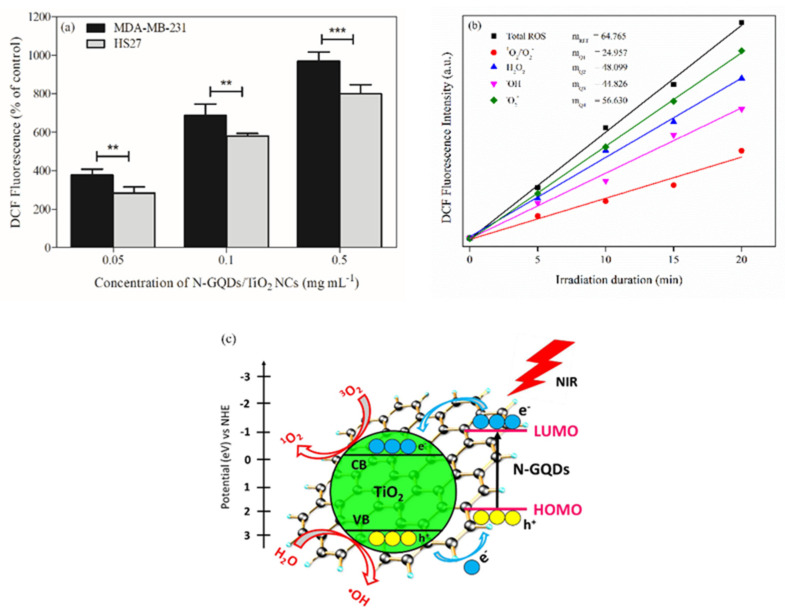
(**a**) ROS levels of MDA-MB-231 and HS27 cells were treated with different concentrations of N-GQDs/TiO_2_ NCs (0.05–0.5 mg mL^−1^) and were irradiated with NIR light for 20 min. Data are presented as the mean ± SD of two independent experiments made in three replicates (*n* = 6) and normalized to control. Significant difference was tested using a two-way ANOVA followed by a Bonferroni post-hoc test and compared to MDA-MB-231, ** (*p* < 0.01) and *** (*p* < 0.001), (**b**) Comparison of photo-induced ROS generated by N-GQDs/TiO_2_ NCs under irradiation of NIR light as a function of irradiation time and (**c**) A proposed possible mechanism of ROS generation by N-GQDs/TiO_2_ NCs upon NIR light irradiation.

The major cause of PDT cytotoxicity is the induction of oxidative stress through the direct production of ROS. The effects of N-GQDs/TiO_2_ NCs as a photosensitizer on ROS generation were evaluated by measuring the intracellular ROS levels using DCFDA staining of the MDA-MB-231 and HS27 cells (Figure 5a). The analysis of the dichlorofluorescein (DCF) intensity indicated that the ROS generated increased as a function of the concentration of nanocomposite (0.05–0.5 mg mL^−1^). The ROS assay carried out provides a clear view of the differential effects of N-GQDs/TiO_2_ NCs-based therapy on MDA-MB-231 and HS27 cells. Minimal fluorescent signals were recorded for HS27 cells. In contrast, MDA-MB-231 registered higher fluorescent intensity, which corresponds with the obtained cell viability of the MDA-MB-231 cells after the PDT treatment. Generally, cancer cells have increased levels of ROS, as they have higher basal ROS levels than in normal cells, which are associated with the abnormal and aggressive growth of cancer cells [60]. However, the reduction–oxidation (redox) balance within cancer cells is maintained by a marked endogenous antioxidant capacity. An excessive amount of ROS can lead to oxidative damage to all components of the cell (lipids, proteins and DNA). Therefore, maintaining ROS homeostasis is vital for cell survival and growth. Contrary to normal/non-cancerous counterparts, cancer cells with increased oxidative stress induced by exogenous agents are more vulnerable to cellular death. This phenomenon reflects the disruption of redox homeostasis, following either elevation of ROS generation or decrease in ROS-scavenging capacity, due to impaired antioxidant system in the cancer cells [61]. Meanwhile, non-cancerous cells with lower levels of basal ROS levels have the capability of maintaining redox homeostasis via the antioxidant defense system, which mainly consists of antioxidants (glutathione) and enzymes (superoxide dismutase, catalase, glutathione peroxidase and glutathione S-transferase). This defense system can scavenge excessive ROS and stabilize the ROS levels under physiological conditions [62,63]. Thus, an increased ROS level was observed in MDA-MB-231 cells when compared to HS27 cells. As the photo-killing effects and intracellular ROS in MDA-MB-231 cells were significantly higher than that of HS27, thus, further study on cellular apoptosis and mitochondrial activity assays were carried out using MDA-MB-231 cells only.

To elucidate a link between oxidative stress and the observed cellular outcomes induced by different PDT doses, four ROS scavengers, sodium pyruvate, DMSO, 1,4-benzoquinone and glycerol as effective scavengers of H_2_O_2_, ^•^OH, O_2_^−•^ and ^1^O_2_/O_2_^−•^ quenchers, were used, respectively. The amount of a particular type of ROS generated by the nanocomposite in the presence of a specific ROS quencher was monitored by the quenching in the fluorescent intensity of DCF. Then, the resulting percentage of ROS was calculated by comparing the decrease in the fluorescent intensity with the measured intensity in the absence of the scavenger (Figure 5b), and the obtained results are listed in Table 1. The calculated results depict that overall, the nature of ROS formed is mainly ^1^O_2_ instead of O_2_^−•^, H_2_O_2_ and ^•^OH, which were responsible for the PDT cytotoxicity that killed the cancer cells. The obtained results were also supported by the reported N-TiO_2_ and ZnO NPs that were applied in PDT as a photosensitizer [35,64]. Besides, based on the literature, it has been stated that singlet oxygen is the major cytotoxic agent that plays a significant role in photobiological activity [65].

Furthermore, Figure 5c shows a proposed possible mechanism of ROS generation by N-GQDs/TiO_2_ NCs upon NIR light irradiation. In the synthesized nanocomposites, N-GQDs with discrete electronic levels serve as a light absorber, generate electrons and enable donor–acceptor contact with TiO_2_, which facilitates direct contact with the TiO_2_ surface. When p-type N-GQDs and n-type TiO_2_ form a p-n heterojunction, free electrons in TiO_2_ are transferred to N-GQDs and thus, create holes in the valence band (VB) of TiO_2_ (Equation (3)) [66]. Upon NIR light irradiation, the N-GQDs absorb the light, leading to the excitation of electrons from the highest occupied molecular orbital (HOMO) to the lowest unoccupied molecular orbital (LUMO), as shown in Equation (4). Then, the electrons are transferred to the CB of TiO_2_ (Equation (5)). Based on the literature, the LUMO level of GQDs is located in the range of −0.5 to −1.0 eV (with respect to normal hydrogen electrode, NHE), and additionally doping with electron-rich N atoms could further lower the work function of the GQDs [67,68]. Moreover, based on our previous work, TiO_2_ NPs synthesized employing the hydrothermal method has a bandgap of 3.00 eV, thus, the position of the conduction band (CB) of anatase TiO_2_ is at −0.18 eV (with respect to NHE), whereby, the LUMO of the N-GQDs is located above the CB of anatase TiO_2_. Therefore, theoretically, the electron transfer from the LUMO of the N-GQDs to CB of the TiO_2_ is thermodynamically favorable, while the generated holes accumulate in the HOMO of N-GQDs. This results in efficient charge separation and greatly suppresses the rate of recombination of e^−^/h^+^ pairs. TiO_2_ accepts the electrons, and the excited electrons reduce the molecular oxygen to generate singlet oxygen molecules (^1^O_2_) (Equations (6) and (7)). According to the energy band positioning profile, the holes in the HOMO (~1.9 eV) is above a threshold of ^•^OH/H_2_O (~2.5 eV). Hence, it would not be able to split the water molecules and produce the hydroxyl radicals (^•^OH) [69]. The holes in the VB of TiO_2_ (2.82 eV) could act as oxidants to oxidize the water molecules into ^•^OH and H^+^ as minor products (Equation (8)). Based on the ROS quenching study, it was found that singlet oxygen molecules are major products formed during the photodynamic process. Therefore, in this case, TiO_2_ served as the catalytic reaction activating region, meanwhile, N-GQDs functioned as a good electron transport medium.

(3) $${\mathrm{TiO}}_{2}\left(e^{-}\right)+N-\mathrm{GQDs}\;\to {\mathrm{TiO}}_{2}\left(h^{+}\right)+N-\mathrm{GQDs}\left(e^{-}\right)$$

(4) $$N-\mathrm{GQDs}+hv\to N-\mathrm{GQDs}\left(e^{-}+h^{+}\right)$$

(5) $$N-\mathrm{GQDs}\left(e^{-}\right)+{\mathrm{TiO}}_{2}\to N-\mathrm{GQDs}\left(h^{+}\right)+{\mathrm{TiO}}_{2}\left(e^{-}\right)$$

(6) TiO2(e−)+O32→TiO2+O2−•

(7) O2−•+H++e−→O12+H2O2

(8) TiO2(h+)+H2O→TiO2+O•H+H+

### 3.6. Mechanism of Photokilling Properties on MDA-MB-231 Cells

**Figure 6:**
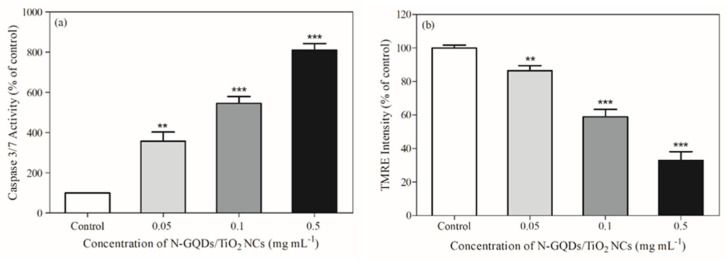
(**a**) Induction of apoptosis, (**b**) Measurement of the mitochondrial membrane potential of MDA-MB-231 cells treated with different concentrations of N-GQDs/TiO_2_ NCs (0.05–0.5 mg mL^−1^) and were irradiated with NIR light for 20 min. Data are presented as the mean ± SD of three replicates (*n* = 3) Significant difference was tested using a one-way ANOVA followed by the Tukey’s post-hoc test as compared to control ** (*p* < 0.01) and *** (*p* < 0.001).

In this work, Caspase Glo-3/7 was employed to study the types of cell death pathways that may occur. Based on Figure 6a, an increase in the nanocomposite concentrations led to an increase in the caspase 3/7 activity and release of caspases. Therefore, successful activation of Caspase Glo-3/7 indicates N-GQDs/TiO_2_-mediated PDT induces the apoptosis-based cell death pathway in MDA-MB-231 cells. The activated executioner caspase (caspase-3 and -7) can result in cleavage of the cellular substrates and eventually leads to the cellular changes observed in apoptotic cells. Once activated, nuclear lamins are cleaved and this is followed by condensation of chromatin and shrinkage of nuclear material [70]. Besides, it also activates the cleavage of Caspase-activated DNase (CAD), which leads to DNA fragmentation [71]. Moreover, cell fragmentation and the formation of apoptotic bodies are caused by cleaved cytoskeletal proteins. This apoptosis signaling pathways are directly involved in inducing cancer cell death.

Generally, apoptosis can be initiated via either activation of death receptors or mitochondrial release of cytochrome c, known as extrinsic and intrinsic apoptosis, respectively [72]. To further study the apoptotic pathway, tetramethylrhodamine ethyl ester (TMRE) was used to label active mitochondria. Based on Figure 6b, exposure to N-GQDs/TiO_2_ NCs in the presence of NIR light irradiation for 20 min altered the mitochondrial membrane potential in the MDA-MB-231 cells by reducing it (decreasing TMRE fluorescence intensity) significantly in a concentration-dependent manner, as accessed with the TMRE assay, which was identified as the early apoptotic signal. Generally, the generated exogenous ROS targets the organelle membrane [73]. The increased production of highly reactive ROS will cause damage to the mitochondrial membranes. This obtained result indicates the collapse of the mitochondrial membrane potential, which triggers mitochondrial outer membrane permeabilization (MOMP). The vast array of cellular stress signals, including MOMP, activates mitochondrial-dependent apoptosis, leading to the release of intermembrane protein, cytochrome c, from the inner membrane of mitochondria into the cytosol, inducing chromatin condensation and the formation of apoptotic bodies [74]. Moreover, the decline in the mitochondria membrane potential level might also be accompanied by the significant drop in adenosine triphosphate (ATP) generation, which consequently leads to initiation of apoptosis (cellular death) due to insufficient energy for cell survival [75]. The findings further imply that exogenous ROS could precede to the mitochondrial dysfunction, which is the critical event of apoptosis, indicating N-GQDs/TiO_2_-mediated PDT induces mitochondrial-dependent apoptosis.

## 4. Conclusions

This work reports the successful synthesis of TiO_2_ conjugated with N-GQDs via the two-pot hydrothermal method through the formation of Ti-O-C on the surface of the TiO_2_. Besides, the light absorption edge of the anatase phase nanocomposite is extended to a longer, non-toxic NIR light region by narrowing down the bandgap to 1.53 eV, which could improve the penetrability of the nanocomposite when administered in deeper locations in the breast tissue. Unlike most metal oxides, the TiO_2_-nanocomposite with N-GQDs did not induce significant toxicity in the absence of light when compared to TiO_2_ NPs, which is a critical property in designing PDT photosensitizers. Under NIR light irradiation, nanocomposite doses (0.1 and 0.5 mg mL^−1^) induced significantly higher cell death in MDA-MB-231 cells than in HS27 cells due to the increased ROS levels, particularly singlet oxygen (^1^O_2_), observed in the cancer cells. The development of this titanium dioxide-based nanocomposite in the current study could be a potential alternative photosensitizer with the ability to effect mitochondrial-dependent apoptosis in the cells. Thus, the incorporation of N-GQDs in TiO_2_ can be a promising candidate for photosensitizer in PDT combined with NIR light activation.

## Figures and Tables

**Table 1:** Proportion of different ROS (%) generated by N-GQDs/TiO_2_ NCs (0.5 mg mL^−1^) under irradiation of NIR light for 20 min.

Types of ROS	Percentage
^1^O_2_/O_2_^−•^	61.5 ± 1.8
H_2_O_2_	25.7 ± 1.7
^•^OH	30.9 ± 0.9
O_2_^−•^	12.6 ± 1.6

Data are presented as the mean ± SD of two independent experiments made in three replicates (*n* = 6).

## Data Availability

Not applicable.

## Supplementary Materials

The following supporting information can be downloaded at: https://www.mdpi.com/article/10.3390/biomedicines10020421/s1, Figure S1: The possible formation mechanism of oxygen vacancies and Ti^3+^ ions; Figure S2: Hydrodynamic size and zeta potential of N-GQDs, TiO_2_ NPs, N-GQDs/TiO_2_ NCs in cell culture medium (mean ± SD, *n* = 3). The nanomaterials were dispersed in water or medium with or without FBS (1%, *v*/*v*), then sonicated, vortexed and hydrodynamic size and zeta; Figure S3: Cell viability of MDA-MB-231 and HS27 cells after 24 h post-treatment. Data are presented as the mean ± SD of two independent experiments made in three replicates (*n* = 6) and normalized to control. Significant difference was tested using two-way ANOVA followed by Bonferroni post-hoc test as compared to control * (*p* < 0.05) and ** (*p* < 0.01); Table S1: Crystallite size, lattice parameters and lattice strain of TiO_2_ NPs and N-GQDs/TiO_2_ NCs.
