# Supplementary material for: N-Doped Graphene Quantum Dots/Titanium Dioxide Nanocomposites: A Study of ROS-Forming Mechanisms, Cytotoxicity and Photodynamic Therapy
Source: Biomedicines. 2022 Feb 10;10(2):421. doi: 10.3390/biomedicines10020421 (PMC8962365; doi:10.3390/biomedicines10020421)
Supplement: Supplementary file 1 [file biomedicines-10-00421-s001.zip › biomedicines-1520064-supplementary.pdf]

Supplementary Material

N-Doped Graphene Quantum Dots/Titanium Dioxide Nanocomposites: A Study of ROS-Forming Mechanisms, Cytotoxicity and Photodynamic Therapy

Pravena Ramachandran [1], Boon-Keat Khor [2], Chong Yew Lee [2], Ruey-An Doong [3], Chern Ein Oon [4], Nguyen Thi Kim Thanh [5,6,]* and Hooi Ling Lee [1,7,]*

[1] Nanomaterials Research Group, School of Chemical Sciences, Universiti Sains Malaysia, USM, Penang 11800, Malaysia; pravena@ioioleo.com
[2] School of Pharmaceutical Sciences, Universiti Sains Malaysia, USM, Penang 11800, Malaysia; khorboonkeat@student.usm.my (B.-K.K.); chongyew@usm.my (C.Y.L.)
[3] Institute of Analytical and Environmental Sciences, National Tsing Hua University, Hsinchu 30013, Taiwan; radoong@mx.nthu.edu.tw
[4] Institute for Research in Molecular Medicine (INFORMM), Universiti Sains Malaysia, USM, Penang 11800, Malaysia; chern.oon@usm.my
[5] Biophysics Group, Department of Physics and Astronomy, University College London, Gower Street, London WC1E 6BT, UK
[6] UCL Healthcare Biomagnetics and Nanomaterials Laboratories, 21 Albemarle Street, London W1S 4BS, UK
[7] School of Chemistry, The University of Sydney, Sydney, NSW 2006, Australia
* Correspondence: ntk.thanh@ucl.ac.uk (N.T.K.T.); hllee@usm.my (H.L.L.)

Table S1. Crystallite size, lattice parameters and lattice strain of TiO_2 NPs and N-GQDs/TiO_2 NCs.

Nanomaterial	Crystallite size (nm)	Lattice parameter, a (Å)	Lattice parameter, c (Å)	Lattice strain
TiO_2 NPs	12.2	3.782	9.514	0.0008
N-GQDs/ TiO_2 NCs	11.2	3.794	9.523	0.0014

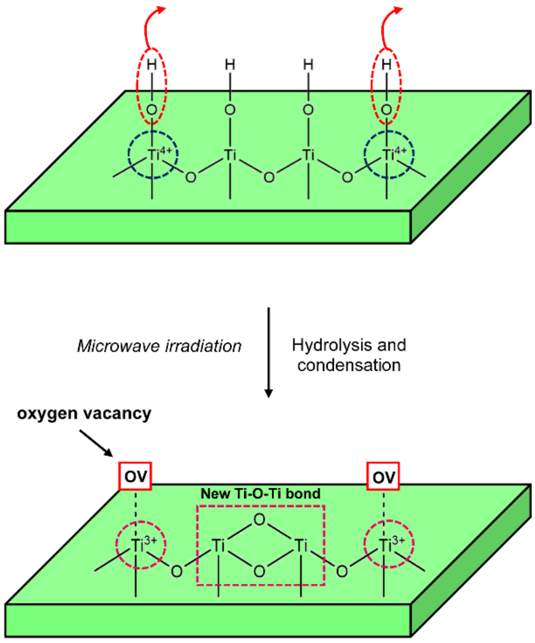

Figure S1 The possible formation mechanism of oxygen vacancies and Ti^{3+} ions.

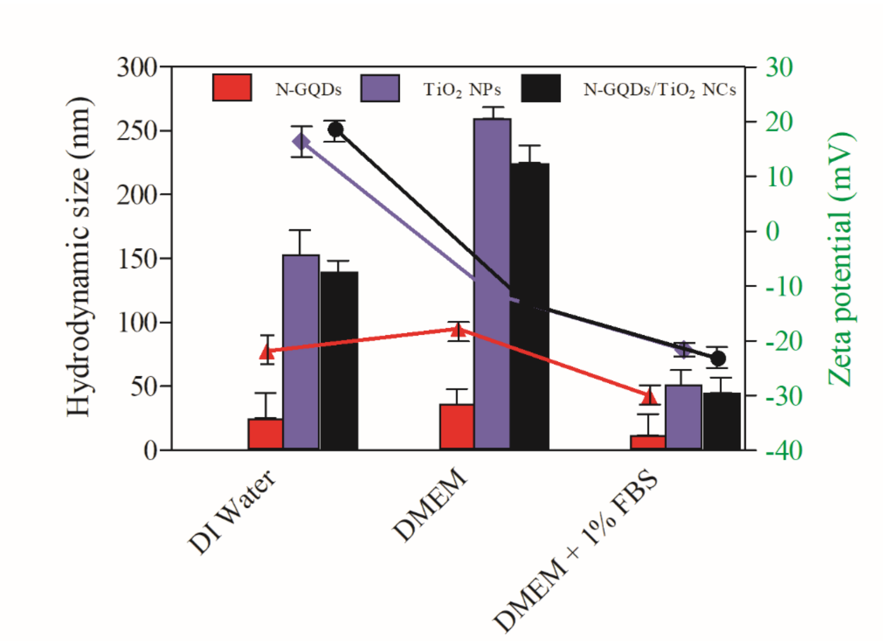

Figure S2 Hydrodynamic size and zeta potential of N-GQDs, TiO_2 NPs, N-GQDs/TiO_2 NCs in cell culture medium (mean ± SD, n=3). The nanomaterials were dispersed in water or medium with or without FBS (1%, v/v), then sonicated, vortexed and hydrodynamic size and zeta

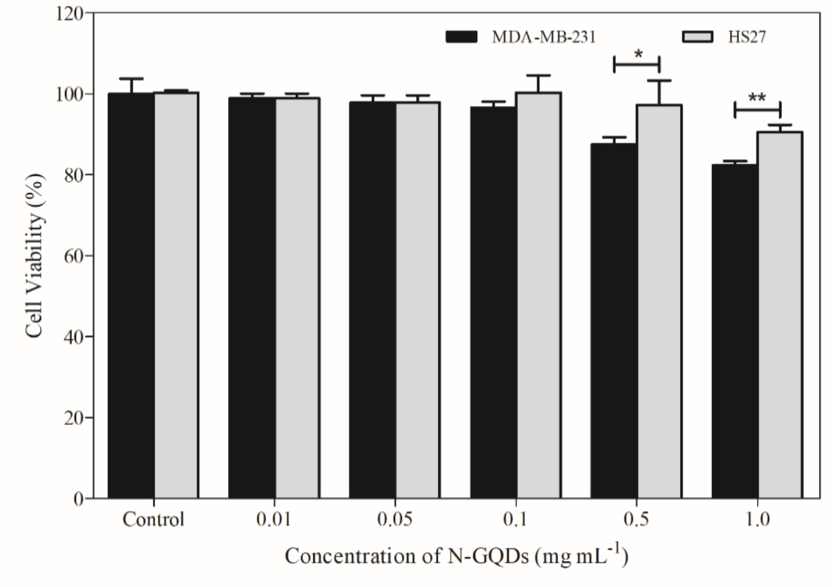

Figure S3 Cell viability of MDA-MB-231 and HS27 cells after 24 h post-treatment. Data are presented as the mean ± SD of two independent experiments made in three replicates (n = 6) and normalized to control. Significant difference was tested using two-way ANOVA followed by Bonferroni post-hoc test as compared to control *($p < 0.05$) and **($p < 0.01$).
